# Supplementary material for: GeneCup: mining PubMed and GWAS catalog for gene–keyword relationships
Source: G3 (Bethesda). 2022 Mar 14;12(5):jkac059. doi: 10.1093/g3journal/jkac059 (PMC9073678; doi:10.1093/g3journal/jkac059)
Supplement: jkac059_Figure_S1 [file jkac059_figure_s1.pdf]

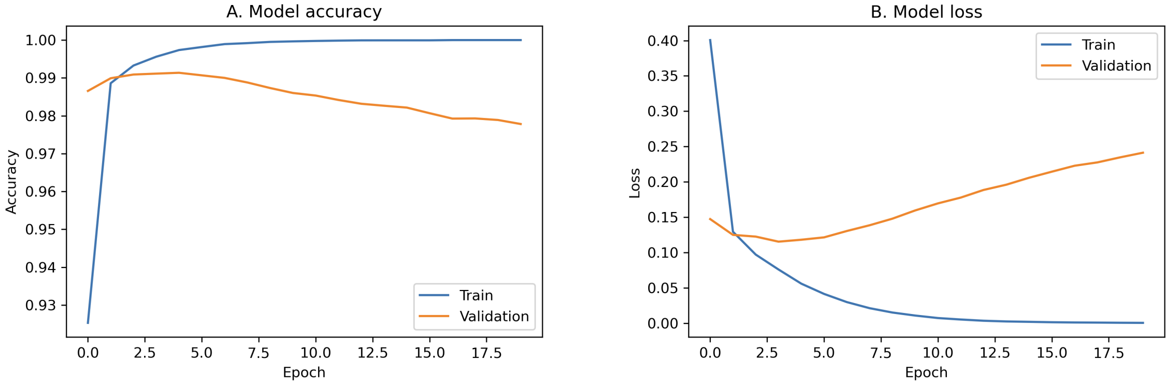

Supplementary Figure 1. The accuracy and loss curve of a convolutional neural network trained to classify sentences containing the word "stress". A convolutional neural network was trained on a dataset containing 19,626 sentences. This dataset was splitted into two parts, having 80% for training and 20% for validation. The gradient based Adamax algorithm was deployed with a learning rate of 0.002 during model training. The accuracy (A) increased rapidly up to 0.995 after the first five epochs. At the same time, the validation accuracy was maximized at 0.991. On the other hand, the loss curve (B) showed a sharp fall followed by a continuous decrease. The increase of the loss curve on the validation set after the fifth epoch was an indication of an overfitting. To avoid overfitting we used the parameters that maximized the validation performance. Our model with these parameters has an AUC of 99.2%.
