# Supplementary material for: GeneCup: mining PubMed and GWAS catalog for gene–keyword relationships
Source: G3 (Bethesda). 2022 Mar 14;12(5):jkac059. doi: 10.1093/g3journal/jkac059 (PMC9073678; doi:10.1093/g3journal/jkac059)
Supplement: jkac059_Figure_S2 [file jkac059_figure_s2.pdf]

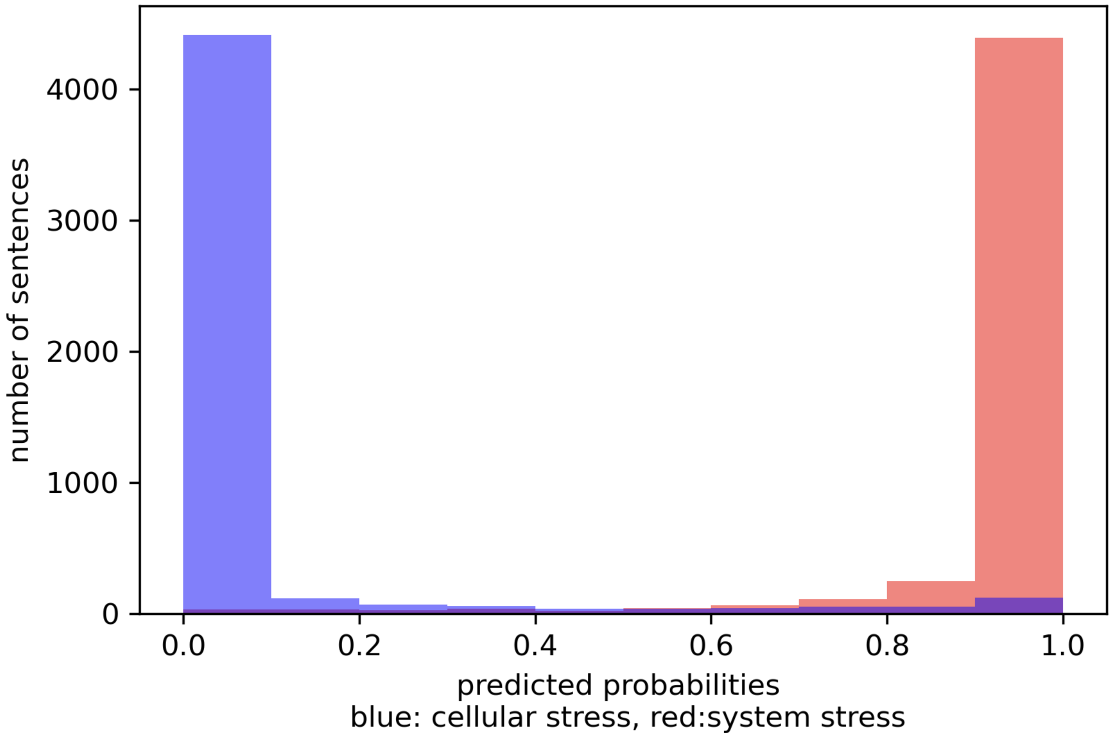

Supplementary Figure 2. Distribution of the predicted probabilities of the test dataset. We tested the convolutional neural network model on a dataset including 5000 sentences from each class containing the cellular stress and system stress related sentences. In order to have a better understanding of the model's reliability of its prediction on the new data, we checked the distribution of the predicted probabilities of the test dataset. The bars represent the number of sentences having the predicted probabilities shown on the x-axis. The sentences having predicted probabilities greater than 0.5 are labelled as systemic stress sentences (red bars). The blue bars represent sentences belonging to the cellular stress class. Among the system stress sentences in the test dataset, 88% of them had predicted probabilities greater than 0.9. Similarly 88% of the cellular stress sentences had predicted probabilities less than 0.1. This indicates that the model has a 90% confidence about the classification of the 88% of the cellular stress sentences.
