# Supplementary material for: GeneCup: mining PubMed and GWAS catalog for gene–keyword relationships
Source: G3 (Bethesda). 2022 Mar 14;12(5):jkac059. doi: 10.1093/g3journal/jkac059 (PMC9073678; doi:10.1093/g3journal/jkac059)
Supplement: jkac059_Figure_S3 [file jkac059_figure_s3.pdf]

chrna7 and stress

9 sentences in 6 studies

Sentence(s) describing systemic stress (classified using a deep learning model):

1. Of these genes, some are critical genes involved in learning and memory, such as cdk5 and **chrna7**, indicating effects of chronic unpredictable **stress** on zebrafish memory. PMID:30718621
2. For duplications, we found that increased expression of **CHRNA7** mRNA is associated with higher expression of nAChR specific and resident ER chaperones, indicating increased ER **stress**. PMID:29129316
3. **Stress** induced IRI protection was absent in **Chrna7** knockout (a7nAChR /) mice and greatly reduced by destroying or transiently inhibiting C1. PMID:28288124
4. The present study examined the association between genetic variation in the nicotinic receptor gene family (CHRNA2, CHRNA3, CHRNA4, CHRNA5, CHRNA6, **CHRNA7**, CHRNA9, CHRNA10, CHRNB2, CHRNB3, CHRNB4) and the occurrence of posttraumatic **stress** disorder (PTSD). PMID:26184988
5. Therefore, the α7 nicotinic acetylcholine receptor gene (**CHRNA7**) may be associated with cortisol **stress** response. PMID:21073885
6. Polymorphisms in the **CHRNA7** promoter were associated with lower cortisol levels after a small laboratory **stress**. PMID:21073885
7. Our findings also show that although the child's **CHRNA7** genotype affects **stress** response, the maternal genotype has a stronger influence on cortisol release after **stress** in male offspring. PMID:21073885

Sentence(s) describing cellular stress (classified using a deep learning model):

1. Interestingly, we showed that deletions in KLF13 and **CHRNA7** influenced the expression of genes belonging to the same immune/inflammatory and oxidative **stress** signaling pathways. PMID:25370694
2. More interestingly, we show that pathways related to immune/inflammatory response and oxidative **stress** signaling are affected by the deletion of KFL13 and **CHRNA7**. PMID:25370694

Supplementary Figure 3. One output page in GeneCup displaying sentences retrieved when querying *chrna7* against the keyword *stress*. The sentences describing stress are classified according to its relation to systemic stress or cellular stress by a convolutional neural network. GeneCup retrieved 9 sentences containing chrna7 and stress from 6 studies. Among them, 7 sentences in 5 studies are predicted as a systemic stress sentence and 2 sentences in 1 study are predicted as cellular stress sentences.
