# Supplementary material for: GeneCup: mining PubMed and GWAS catalog for gene–keyword relationships
Source: G3 (Bethesda). 2022 Mar 14;12(5):jkac059. doi: 10.1093/g3journal/jkac059 (PMC9073678; doi:10.1093/g3journal/jkac059)
Supplement: jkac059_Table_S1 [file jkac059_table_s1.pdf]

Supplementary Table 1. Mini ontology for addiction related concepts

Category	Keywords	Terms
Addiction	addiction	addiction, addictive, compulsive, drug-abuse, escalation, punishment
	aversion	aversion, aversive, conditioned taste aversion, CTA
	dependence	dependence
	intoxication	binge, intoxication
	relapse	craving, drug seeking, reinstatement, relapse, seeking
	reward	conditioned place preference, CPP, drug reinforced, hedonic, ICSS, incentive, instrumental response, intracranial self-stimulation, operant, reinforcement, reinforcing, reward, self-administered, self-administration
	sensitization	sensitization
	withdrawal	withdrawal
Brain	accumbens	acbc, acbs, accumbal, accumbens, core, Nacc, NacSh, shell
	amygdala	amy, amygdala, bla, cea, cna
	cortex	cerebral, cingulate, cortex, cortico limbic, corticolimbic, corticostriatal, infralimbic, insula, insular, mPFC, orbitofrontal, pfc, prefrontal, prelimbic, prl, vmpfc
	habenula	habenula, lhb, mhb
	hippocampus	ca1, ca3, dentate gyrus, dhpc, hip, hipp, hippocampal, hippocampus, subiculum, vhipp, vhpc
	hypothalamus	hypothalamic, hypothalamus, LHA, paraventricular nucleus, PVN
	striatum	basal ganglia, caudate, globus pallidus, GPI, putamen, STR, striatal, striatum
	VTA	limbic, mesoaccumbal, mesoaccumbens, mesolimbic, midbrain, pvta, ventral tegmental, vta
	alcohol	acamprosate, alcohol, alcoholic, alcoholics, alcoholism, antabuse, campral, disulfiram, ethanol, naltrexone, revia, vivitrol
	amphetamine	AMPH, amphetamine, METH, methamphetamine
	benzodiazepine	adinazolam, alprazolam, benzodiazepine, benzos, brotizolam, chlordiazepoxide, climazolam, clobazam, clonazepam, clorazepate, diazepam, estazolam, flunitrazepam,

Drugs		flurazepam, halazepam, librium, loprazolam, lorazepam, lormetazepam, midazolam, nimetazepam, nitrazepam, normison, oxazepam, prazepam, temazepam, triazolam, valium, xanax
	cannabinoid	acylethanolamines, cannabichromene, cannabidiol, cannabigerol, cannabinoid, cannabinoids, cannabinol, cannabis, cannabivarin, cesamet, drobinal, dronabinol, endocannabinoid, endocannabinoids, epidiolex, JWH-018, JWH-122, JWH-250, marijuana, marinol, nabilone, Oleoylethanolamide, palmitoylethanolamide, phytocannabinoid, rimonabant, SR141716, SR144528, syndros, tetrahydrocannabinol, tetrahydrocannabivarin, thc, thc-9
	cocaine	cocaine
	nicotine	nicotine, smoker, smokers, smoking, tobacco
	opioid	buprenorphine, codeine, fentanyl, heroin, hycodan, hydrocodone, hydromorphone, kadian, kratom, methadone, morphine, naloxone, opioid, opioids, oxycodone, oxycontin, percocet, suboxone, tramadol, ultram, vicodin
	psychedelics	ayahuasca, ecstasy, ibogaine, ketamine, LSD, lysergic acid diethylamide, MDMA, mescaline, methylenedioxymethamphetamine, N-methoxybenzyl, NBOMe, NBOMes, peyote, psilocybin, psychedelic, psychedelics
Molecular function	neuroplasticity	boutons, epsc, epsp, IPSC, IPSP, long term depression, long term potentiation, LTD, LTP, mIPSC, neurite, neurogenesis, neuroplasticity, plasticity, synaptic
	neurotransmission	5-ht, acetylcholine, cholinergic, DAergic, dopamine, dopaminergic, GABA, GABAergic, glutamate, glutamatergic, muscarinic, neuropeptide, neuropeptides, neurotransmission, nicotinic, serotonergic, serotonin
	signalling	glycosylation, phosphorylation, signaling, signalling, kinase, binding, signal transduction, second messengers, cGMP, cAMP
	transcription	histone, hypermethylation, hypomethylation, methylation, ribosome, transcription
Psychiatric diseases	anxiety	anxiety, anxious
	autism	autism, autistic
	bipolar	bipolar disorder
	compulsive	compulsive, obsessive

	depression	depression, depressive, major depressive disorder, MDD
	impulsivity	5-CSRTT, 5-choice task, delay discounting, delay exposure, delay intolerance, delayed reward, delay task, five choice serial reaction time task, impulsive, impulsivity, premature responding
	schizophrenia	schizophrenia
Cell	neuron	adrenergic neuron, adrenergic neurons, cholinergic neuron, cholinergic neurons, dopaminergic neuron, dopaminergic neurons, gabaergic neuron, gabaergic neurons, glutamatergic neuron, glutamatergic neurons, GnRH neuron, GnRH neurons, interneuron, interneurons, monoaminergic neuron, monoaminergic neurons, medium spiny neuron, medium spiny neurons, motor neuron, motor neurons, neuronal cell, neuronal cells, nitrergic neuron, nitrergic neurons, noradrenergic neuron, noradrenergic neurons, projection neuron, projection neurons, pyramidal neuron, pyramidal neurons, sensory neuron, sensory neurons, serotonergic neuron, serotonergic neurons, somatostatin neuron, somatostatin neurons, neuron, neurons, excitatory neuron, excitatory neurons, inhibitory neuron, inhibitory neurons, corticospinal neuron, corticospinal neurons, dopamine neuron, dopamine neurons, D1 neuron, D1 neurons, afferent neuron, afferent neurons, efferent neuron, efferent neurons, serotonin neuron, serotonin neurons, cortical neuron, cortical neurons, hippocampal neuron, hippocampal neurons, DA neuron, DA neurons, CNS neuron, CNS neurons, cortex neuron, cortex neurons, mesencephalic neuron, mesencephalic neurons, orexin neuron, orexin neurons, catecholaminergic neuron, catecholaminergic neurons, striatal neuron,striatal neurons
	astrocyte	astrocyte, astrocytes, astrocytic, astroglia, astroglial
	microglia	microglia, microglial
	endothelium	endothelium, endothelial cell, endothelial cells
	oligodendrocyte	oligodendrocyte, oligodendrocytes
Stress	PTSD	PTSD, post-traumatic stress, post-traumatic stress symptoms, post-traumatic stress disorder
	stress	distress, psychological trauma, stress
